# Design of pH Sensitive Binding Proteins from the Hyperthermophilic Sso7d Scaffold

**DOI:** 10.1371/journal.pone.0048928

**Published:** 2012-11-07

**Authors:** Nimish Gera, Andrew B. Hill, Dalon P. White, Ruben G. Carbonell, Balaji M. Rao

**Affiliations:** Department of Chemical and Biomolecular Engineering, North Carolina State University, Raleigh, North Carolina, United States of America; Cleveland Clinic Lerner Research Institute, United States of America

## Abstract

We have engineered pH sensitive binding proteins for the Fc portion of human immunoglobulin G (hIgG) (hFc) using two different strategies – histidine scanning and random mutagenesis. We obtained an hFc-binding protein, Sso7d-hFc, through mutagenesis of the Sso7d protein from the hyperthermophilic archaeon *Sulfolobus solfataricus*; Sso7d-hFc was isolated from a combinatorial library of Sso7d mutants using yeast surface display. Subsequently, we identified a pH sensitive mutant, Sso7d-his-hFc, through systematic evaluation of Sso7d-hFc mutants containing single histidine substitutions. In parallel, we also developed a yeast display screening strategy to isolate a different pH sensitive hFc binder, Sso7d-ev-hFc, from a library of mutants obtained by random mutagenesis of a pool of hFc binders. In contrast to Sso7d-hFc, both Sso7d-his-hFc and Sso7d-ev-hFc have a higher binding affinity for hFc at pH 7.4 than at pH 4.5. The Sso7d-mutant hFc binders can be recombinantly expressed at high yield in *E. coli* and are monomeric in solution. They bind an epitope in the CH3 domain of hFc that has high sequence homology in all four hIgG isotypes (hIgG_1–4_), and recognize hIgG_1–4 _as well as deglycosylated hIgG in western blotting assays. pH sensitive hFc binders are attractive candidates for use in chromatography, to achieve elution of IgG under milder pH conditions. However, the surface density of immobilized hFc binders, as well as the avidity effect arising from the multivalent interaction of dimeric hFc with the capture surface, influences the pH dependence of dissociation from the capture surface. Therefore, further studies are needed to evaluate if the Sso7d mutants identified in this study are indeed useful as affinity ligands in chromatography.

## Introduction

The affinity and specificity of protein-protein interactions can be regulated by external pH. Indeed, sensitivity of binding affinity to pH plays an important role in biological processes. For instance, maternal immunoglobulin G (IgG) binds the neonatal Fc receptor (FcRn) with high affinity at pH 6.0 and weakly at pH 7.4. This pH sensitivity of binding facilitates transcytosis of maternal IgG across fetal and neonatal tissues and is critical for imparting passive immunity to the fetus before a functional immune system is developed [1], [2]. The introduction of pH sensitive binding activity can be also used to increase the potency of therapeutic proteins [3]–[5]. Binding of a protein to its target receptor typically results in internalization of the receptor-protein complex, and subsequent degradation in the endosome. Therapeutic proteins that are engineered to lose binding to their target receptor in the acidic environment of the endosome (pH 6.0) can escape endosomal degradation and result in increased half-life of the protein in the extracellular space. This paradigm has been used to engineer pH sensitive mutants of Granulocyte Colony Stimulating Factor (GCSF) [5] and therapeutic antibodies against the Interleukin-6 receptor (IL-6R) and Proprotein Convertase Substilisin Kexin type 9 (PCSK9) [3], [4].

Introduction of one or more histidine residues in the binding interface is commonly used to engineer pH sensitivity of binding. The protonation of the histidine side chain changes at lower pH, thereby altering the electrostatic interactions involved in binding, and leads to a change in binding affinity. A computational approach can be used to identify specific residues in the binding interface to be mutated to histidine [5]. Alternatively, histidine scanning mutagenesis of putative binding interfaces, such as complementarity determining regions (CDRs) in antibodies, maybe used to identify histidine substitutions that result in pH sensitivity of binding [3], [4]. Phage display has been used to efficiently screen a combinatorial library of histidine mutants to identify a pH sensitive single domain antibody [6]. While histidine residues in the binding interface mediate pH sensitivity through their electrostatic interactions, introduction of ionizable residues in the protein core may also lead to pH-dependent conformational changes, and therefore, pH sensitivity of binding [7].

In this study, we engineered pH sensitive binding proteins for a model target – the Fc portion of human IgG (hIgG) (hFc) – using two different strategies: histidine scanning and random mutagenesis. We have previously shown that the Sso7d protein from the hyperthermophilic archaeon *Sulfolobus solfataricus* is a versatile scaffold for generating binding proteins for a wide spectrum of targets [8]. Here we isolated an hFc-binding protein, Sso7d-hFc, from a library of Sso7d mutants. Subsequently, we systematically evaluated the pH sensitivity of binding of Sso7d-hFc mutants containing single histidine substitutions, and identified a pH sensitive hFc binder (Sso7d-his-hFc). In parallel, we also developed a yeast-display based screening strategy to isolate a pH sensitive binder, Sso7d-ev-hFc, from a library obtained by random mutagenesis of a pool of Sso7d-based hFc binders. Notably, unlike Sso7d-hFc, both Sso7d-his-hFc and Sso7d-ev-hFc have a higher binding affinity for hFc at pH 7.4 than at pH 4.5.

## Materials and Methods

### Isolation of hFc Binders from a Library of Sso7d Mutants

The Sso7d library described previously [8] was screened using magnetic selection and fluorescence activated cell sorting (FACS) as described [9], [10]. Briefly, yeast cells grown in SDCAA medium (20 g/L dextrose, 5 g/L casamino acids, 6.7 g/L yeast nitrogen base, 5.40 g/L Na_2_HPO_4_, 7.45 g/L NaH_2_PO_4_) were passaged into SGCAA medium (20 g/L galactose, 5 g/L casamino acids, 6.7 g/L yeast nitrogen base, 5.40 g/L Na_2_HPO_4_, 7.45 g/L NaH_2_PO_4_) to obtain a cell density of 10^7^ cells/ml. Cells were cultured in SGCAA at 20°C and 250 rpm for 20–24 hours to induce protein expression on the yeast cell surface. 100 µl of Dynal™ biotin binder beads (4×10^8^ beads/ml; Invitrogen, Carlsbad, CA) were pre-coated with biotinylated hFc protein (Jackson Immunoresearch, Westgrove, PA) overnight at 4°C. 2×10^9^ cells (20X library diversity) were incubated with biotin binder beads for negative selection for 1 hour at 4°C. Bead-bound cells were discarded and further negative selection was performed against mouse IgG (mIgG), chicken immunoglobulin Y (cIgY) and rabbit IgG (rIgG). Finally, unbound cells were used for a positive selection against hFc-coated beads for 1 hour at 4°C. The bead-bound cells were washed four times with PBS-BSA (8 g/L NaCl, 0.2 g/L KCl, 1.44 g/L Na_2_HPO_4_, 0.24 g/L KH_2_PO_4_, pH 7.4 containing 0.1% BSA), resuspended in SDCAA and grown for 24–48 hours at 30°C and 250 rpm. All selections were done in the presence of PBS-BSA at pH 7.4.

Cell surface protein expression was induced again in the pool of cells obtained after magnetic selection, by culturing cells at a starting cell density of 10^7^/ml in SGCAA medium for 24 hours. FACS was performed to select for the highest affinity binders for Sso7d-hFc on a FACS Aria (Becton Dickinson, San Jose, CA) flow cytometer, as described [10]. Cells were simultaneously labeled with an anti-c-myc chicken antibody (Invitrogen, Carlsbad, CA) and biotinylated hFc. A goat anti-chicken antibody conjugated to Alexa Fluor-633 and streptavidin-phycoerythrin (strep-PE) (Invitrogen, Carlsbad, CA) were used as secondary reagents. After four rounds of FACS, cells were plated on SDCAA plates (20 g/L dextrose, 5 g/L casamino acids, 6.7 g/L yeast nitrogen base, 182 g/L sorbitol, 5.40 g/L Na_2_HPO_4_, 7.45 g/L NaH_2_PO_4,_ 15 g/L Agar) and clones were picked for sequencing.

### End Point Assay for pH Sensitivity

Yeast cells displaying Sso7d mutants were incubated with 100 nM hFc-biotin (for Sso7d-hFc and Sso7d-his-hFc) or 2 µM hFc-biotin (for Sso7d-ev-hFc) in P1-BSA (100 mM sodium phosphate buffer pH 7.4, 0.1% BSA) for 15 min at 4°C. Subsequently, labeled cells were washed with P1-BSA and incubated with 1 ml of P2-BSA (100 mM sodium phosphate buffer pH 4.5, 0.1% BSA) with shaking at room temperature. A control sample, where the cells were incubated in 1 ml of P1-BSA was also prepared. After 30 min, cells were labeled with strep-PE and analyzed on a BD FACS Aria flow cytometer.

### Histidine Scanning

Two fragments from the Sso7d-hFc gene were amplified by PCR, using internal primers that introduced the histidine mutation one by one at the ten mutated amino acid residues in Sso7d-hFc. The two PCR fragments were designed to share 30–40 bp homology with each other, and 50 bp homology with the pCTCON vector used for yeast surface display. Linearized pCTCON vector was prepared by digestion with *NheI* and *BamHI* restriction enzymes (New England Biolabs (NEB), Ipswich, MA). Subsequently, homologous recombination in yeast was used to assemble the complete gene for each histidine scanning mutant. This is the same protocol that is used for construction of yeast display libraries using multiple gene fragments [10]. The identity of the mutants was confirmed by DNA sequencing.

### Random Mutagenesis Library Construction and Cell Sorting

Plasmid DNA was isolated using Zymoprep Kit II (Zymoresearch Corporation, Orange, CA), from the pool of hFc binders after magnetic selection and a higher affinity pool after two round of FACS, obtained during the process of isolating Sso7d-hFc. Error prone PCRs were performed on DNA isolated from each pool, using the nucleotide analogues 8-oxo-dGTP and dPTP (Trilink Biotechnologies, San Diego, CA) as described [11]–[13]. Four different combinations with varying nucleotide analogue concentrations and number of PCR cycles were used: 10 cycles with 10 µM each of 8-oxo-dGTP and dPTP, 20 cycles with 10 µM each of 8-oxo-dGTP and dPTP, 20 cycles with 2 µM each of 8-oxo-dGTP and dPTP, 30 cycles with 2 µM each of 8-oxo-dGTP and dPTP. A 50 µl PCR reaction mixture consisted of 1X Taq Buffer without MgCl_2_, 2 mM MgCl_2_, primers Pf1 and Pr1 at 0.1 µM each, 200 µM dNTPs, 20 ng template DNA and 0.05 U/µl of Taq DNA polymerase (Invitrogen, Carlsbad, CA).The primer sequences were: Pf1 - 5′AGT GGT GGT GGT GGT TCT GGT GGT GGT GGT TCT GGT GGT GGT GGT TCT GCT AGC ATG GCG ACC GTG AAA TTT AAA TAT AAA G 3′ and Pr1 - 5′CTC GAG CTA TTA CAA GTC CTC TTC AGA AAT AAG CTT TTG TTC GGA TCC TTT TTT CTG TTT TTC CAG CAT CTG 3′. All primers were obtained from Integrated DNA Technologies (IDT; Coralville, IA). The mixture was denatured at 94°C for 3 min followed by 10, 20 or 30 cycles of 94°C for 45 s, 58°C for 30 s, 72°C for 90 s and a final extension step at 72°C for 10 min. The PCR products were purified using the Qiagen PCR purification kit (Qiagen, Valencia, CA). Subsequently, 200 ng of each product was mixed to create a combined DNA pool from all different reactions. This mixed product was further used as a template for PCR amplification. The reaction mix consisted of 1X high fidelity Phusion buffer, 0.1 µM primers, 200 µM dNTPs and Phusion high fidelity DNA polymerase (1U/50 µl, NEB). PCR was performed with an initial denaturation at 98°C for 2 min and 30 cycles of 98°C for 1 min, 61°C for 30 s, 72°C for 90 s and a final extension step at 72°C for 10 min. PCR products were concentrated using Pellet Paint™ (Novagen, San Diego, CA).

Homologous recombination mediated gap repair was used to generate a yeast surface display library. 1 µg of linearized pCTCON plasmid with 3 µg of insert was transformed in *Sacchromyces cerevisiae* strain EBY100 as described [8], [10]. The electroporation was performed using a Bio-Rad Gene Pulser X cell system (Bio-Rad, Hercules, CA) at 0.54 kV, 25 µF and 1000 Ω. Transformed EBY 100 cells were grown in YPD medium (10 g/L yeast extract, 20 g/L peptone, 20 g/L dextrose) for 1 hour at 30°C and 250 rpm. Serial dilutions were plated on SDCAA plates to estimate the library diversity as ∼ 10^7^ mutants. The rest of the library was grown in SDCAA medium with 1∶100 pen-strep solution (Invitrogen, Carlsbad, CA) for 24–48 hours. The library was passaged twice in SDCAA medium before proceeding to magnetic selection.

Magnetic selection was conducted as described earlier and included a negative selection step with biotin binder beads and positive selection with hFc-biotin. The bead-bound cells were resuspended in P2-BSA and incubated for 30 min with shaking at room temperature. The cells in the supernatant were further expanded in SDCAA medium for 48 hours at 30°C and 250 rpm. These cells were further sorted by FACS. Two sorts for higher affinity were performed with 2 µM hFc-biotin labeling in P1-BSA. The sorted population was labeled with 100 nM hFc-biotin in P2-BSA and cells that lost binding to hFc-biotin were selected. Subsequently, two additional end-point sorts were performed, where cells were incubated with 2 µM hFc-biotin, in P1-BSA for 15 min at 4°C, following which yeast-hFc complexes were dissociated in P2-BSA for 30 min at room temperature with shaking. Mutants that lost binding to hFc-biotin were selected. Cells from the final sort were plated on SDCAA plates and individual clones were picked for sequencing. Plasmid DNA was isolated from the Sso7d mutants using Zymoprep Kit II. The isolated DNA was further transformed into Novablue™ (*E. coli*) cells (EMD Biosciences, San Diego, CA). A Qiagen miniprep kit was used to isolate plasmids from *E.coli* (Qiagen, Valencia, CA) and sequenced using the primer 5′ ACT ACG CTC TGC AGG CTA GT 3′.

### Specificity Analysis

Yeast cells expressing Sso7d-hFc, Sso7d-his-hFc and Sso7d-ev-hFc as cell surface fusions were labeled with 1 µM hFc, mIgG, cIgY, rIgG, goat-IgG (gIgG), donkey IgG (dIgG), Fab and Fab2 (Jackson Immunoresearch, Westgrove, PA) and analyzed by flow cytometry. All proteins were biotinylated and were detected using strep-PE.

### Recombinant Expression and Purification of Sso7d Mutants

Sso7d mutants were cloned into the pET22b(+) vector, as previously described [8]. One liter of 2XYT medium was inoculated with a 5 ml overnight culture of Rosetta Cells™ (EMD Biosciences, San Diego, CA), harboring the corresponding plasmid for the Sso7d mutant. When cells in culture reached an OD_600_ of 0.7, recombinant protein expression was induced with 0.5 mM of IPTG and cells were grown for another 19–20 hours at 37°C and 250 rpm. The cells were harvested by centrifugation at 4700 rpm for 20 min and supernatant was discarded. The cell pellet was resuspended in Buffer A (50 mM Tris pH 7.4, 300 mM NaCl) with 2 mM phenylmethylsulfonyl fluoride. Cell lysis was performed by sonication for 10 min. The lysed cells were centrifuged at 15000 rpm and the supernatant was filtered with a 0.22 µm filter. The filtered sample was loaded onto a 5 ml Bio-Scale Mini Profinity™ IMAC Cartridge (Bio-Rad, Hercules, CA) and purified on a Bio-rad Biologic LP system. Sso7d variants containing a 6x-histidine (6x-his) tag were eluted using a linear gradient of Buffer A and Buffer B (50 mM Tris pH 7.6, 300 mM NaCl, 500 mM Imidazole). The collected fractions were analyzed by SDS-PAGE analysis using Novex 10% Bis-Tris Gels (Invitrogen, Carlsbad, CA) and the pure protein fractions were pooled together. The purified proteins were dialyzed in 50 mM sodium phosphate buffer with 300 mM NaCl at pH 7.4 or pH 4.5. Protein concentrations were measured using bicinchoninic acid (BCA) assay (Thermo scientific, Rockford, IL) with bovine serum albumin (BSA) as a standard. Estimates of purified protein yield ranged from 40–50 mg per liter of bacterial culture. Analytical size exclusion chromatography experiments were performed to confirm the oligomeric state of the binding proteins at pH 7.4 and 4.5.

### Western Blotting Analysis

Purified forms of the four human IgG isotypes- hIgG_1_, hIgG_2_, hIgG_3_ and hIgG4 (Sigma Aldrich, St. Louis, MO), the composite hIgG (Equitech bio, Kerrville, TX), hIgG digested with PNGase F (NEB) and an undigested hIgG control were run on 8% SDS-PAGE gels using standard procedures. PNGase F digestion was performed according to the manufacturer’s protocol. Subsequently, the proteins were transferred onto a PVDF membrane (GE Healthcare Bio-Sciences, Piscataway, NJ) using a semi-dry blotting unit (Fisher Scientific, Pittsburgh, PA) with 1X Transfer buffer (25 mM Tris, 192 mM glycine, 10% methanol, 0.5% SDS). The membrane was blocked with 5% non-fat dry milk (Lab scientific, Livingston, NJ) in 1X TBS-T (0.01 M Tris-HCl, 0.15 M NaCl, 0.05% Tween-20). Sso7d-hFc, Sso7d-his-hFc and Sso7d-ev-hFc were biotinylated using the EZ-Link Sulfo-NHS-LC biotinylation kit (Thermo Scientific, Rockford, IL) and used as primary reagents for detecting IgG in western blotting analysis. The membrane was incubated with the biotinylated proteins overnight at 4°C. Secondary labeling was performed using an anti-biotin-HRP conjugated antibody (Cell Signaling Technology Inc., Danvers, MA) for 1 hour. The blot was developed using the SuperSignal West Femto Chemiluminescent Substrate (Thermo Scientific, Rockford, IL) and imaged on a Bio-Rad chemiluminescence Imager.

### Circular Dichroism (CD) Analysis

Soluble proteins Sso7d-hFc, Sso7d-his-hFc and Sso7d-ev-hFc were dialyzed in 50 mM sodium phosphate buffer with 150 mM Na_2_SO_4_ to avoid background signal from chloride ions in CD spectra. Protein samples were analyzed on a JASCO-815 spectropolarimeter to record CD spectra over the range of wavelengths 210–240 nm, at 50 nm/min, 0.1 nm pitch, 1 nm bandwidth and 2 s response time. Three accumulation scans were done for each sample. Baseline-subtracted molar ellipticity values (θ) were normalized as:
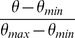
where θ_min_ and θ_max_ are the minimum and maximum values of baseline-subtracted molar ellipticity.

### Estimation of K_D_ Using Enzyme Linked Immunosorbent Assay (ELISA)

The K_D_ of the binding interaction between hFc and Sso7d mutants at pH 7.4 and pH 4.5 was measured using ELISA, as previously described [14]. 100 µl per well of hIgG (1 µg/ml for measurements with Sso7d-hFc and 2 µg/ml for Sso7d-his-hFc and Sso7d-ev-hFc) in 1X phosphate buffered saline(PBS) was immobilized on a 96-well microtiter plate (Nunc Medisorp, Thermo Scientific, Rockford, IL) overnight at 4°C with shaking. The wells were washed five times with 200 µl of wash buffer (PBS +0.05% Tween-20) and blocked for 4 hours with blocking buffer (PBS+0.05% Tween 20+1% BSA) at room temperature. After washing five times with wash buffer, wells were incubated with soluble Sso7d-hFc (0–1.5 µM), Sso7d-his-hFc (0–3 µM) and Sso7d-ev-hFc (0–20 µM) at pH 7.4 overnight at 4°C with shaking. Alternately, Sso7d-hFc (0–1.5 µM), Sso7d-his-hFc (0–30 µM) and Sso7d-ev-hFc (0–20 µM) were incubated at pH 4.5 in 50 mM sodium phosphate and 300 mM NaCl. The wells were washed five times and incubated with 100 µl of a 1∶2000 dilution of mouse anti-his-alkaline phosphatase conjugated antibody (Abcam, Cambridge, MA) in blocking buffer for 1 hour at 4°C. Wells were washed five times and incubated with 100 µl of p-nitrophenyl phosphate substrate (Sigma Aldrich, St. Louis, MO). The absorbance was read in a microplate reader (Perkin Elmer, Waltham, MA) at 405 nm. Triplicate wells coated with hIgG were used for each protein concentration; triplicate wells coated with 100 µl 6% BSA were used for background subtraction. All absorbance values were normalized to maximum absorbance and data from at least two different experiments were globally fit to a 4-parameter logistic model [15] using non-linear least squares regression to estimate the K_D_. The error associated with the K_D_ estimate was calculated as previously described [10].

### Epitope Mapping Using Yeast Surface Display

hFc was sub-cloned into the yeast display (pCTCON) vector from an hFc-containing plasmid that was a kind gift from Dr. Jeffery Yoder at NCSU. Expression of hFc as a yeast cell surface fusion was confirmed by flow cytometry using an anti-c-myc chicken antibody (Invitrogen, Carlsbad, CA) followed by secondary labeling using a goat-anti-chicken-Alexa-Fluor-633 antibody (Invitrogen, Carlsbad, CA). Binding to Alexa-Fluor 488 conjugated Protein A (Invitrogen, Carlsbad, CA) was used to confirm proper folding of hFc on the yeast cell surface. Heat denaturation of yeast displayed hFc was performed by heating 10^6^ cells to 99°C in a PCR machine. Subsequently, cells were chilled on ice for 30 min and labeled with soluble Sso7d-hFc (2 µM), followed by a penta-his-Alexa-Fluor-647 (Qiagen, Valencia, CA) secondary antibody for analysis by flow cytometry.

Error-prone PCR was used to generate a yeast surface display library of hFc mutants. Genemorph II random mutagenesis kit (Agilent, Santa Clara, CA) was used to generate a library with a low mutagenesis rate. pCTCON plasmid containing the gene for hFc was used as a template for PCR. The following primers were used: Pf2-5′- AGT GGT GGT GGT GGT TCT GGT GGT GGT GGT TCT GGT GGT GGT GGT TCT GCT AGC CCC AAA TCT TGT GAC AAA ACT -3′ and Pr2-5′- CTC GAG CTA TTA CAA GTC CTC TTC AGA AAT AAG CTT TTG TTC GGA TCC TTT ACC CGG AGA CAG GGA -3′. The DNA library (12 µg) was transformed along with 4 µg linearized pCTCON vector (digested using *NheI*, *BamHI* and *SalI*) into yeast EBY100, as described earlier. Serial dilutions on SDCAA plates were used to determine the library diversity. The library was passaged twice before inducing expression in SGCAA medium for 24 hours at 20°C. The hFc library was labeled with 2 µM Sso7d-hFc and the anti-cmyc antibody, and three rounds of FACS were used to identify mutants with loss of binding to Sso7d-hFc. Secondary detection was obtained by penta-his-Alexa-Fluor-647 and goat-anti-chicken-Alexa-Fluor-633. After the final sort, cells were plated on SDCAA plates and individual clones were picked for DNA sequencing and further analysis by flow cytometry.

### pH Sensitivity of hFc-binding to Sso7d Mutants Immobilized on a Surface

A C-terminal cysteine was introduced in all mutants (Sso7d-hFc, Sso7d-his-hFc and Sso7d-ev-hFc) using standard molecular cloning techniques and recombinant proteins were expressed and purified as described earlier. Proteins were eluted with a gradient of Buffer A and Buffer B containing 2 mM dithiothreitol. Purified proteins were dialyzed in 50 mM sodium phosphate buffer with 300 mM NaCl at pH 7.4.

Maleimide-activated microtiter plates (Thermo scientific, Rockford, IL) were washed three times with 200 µl wash buffer (0.1 M sodium phosphate, 0.15 M NaCl, 0.05% Tween-20 Detergent; pH 7.0). Treatment with tris (2-carboxyethyl) phosphine (TCEP) (Thermo scientific, Rockford, IL) was used to ensure that the proteins contained a reduced form of cysteine, right before the assay. Reduced cysteine-tagged mutants (2 µg/ml) in binding buffer (0.1 M sodium phosphate, 0.15 M NaCl, 10 mM EDTA, pH 7.0), were added to the wells of the washed plate and incubated overnight with shaking at 4°C. The wells were washed three times with wash buffer and blocked with 10 µg/ml cysteine (Sigma-Aldrich, St. Louis, MO) for 1 hour at room temperature. Wells were washed thrice with 200 µl wash buffer and incubated with shaking in pH 2.5 buffer (10 mM sodium acetate, pH 2.5) for 30 min at room temperature. All wells were incubated with 6 µM of biotinylated hIgG (except for the control wells with no IgG) for 1 hour at room temperature. After washing the wells thrice, buffers of the following different pH values were used to dissociate hIgG: pH 7.4 and pH 4.5 (50 mM sodium phosphate, 300 mM NaCl), pH 3.5 and pH 2.5 (10 mM sodium acetate). All dissociation buffers had 5 mg/ml of unbiotinylated hIgG and the dissociation was carried out for 30 min at room temperature. The wells were washed three times with wash buffer and a 1∶2000 dilution of streptavidin-alkaline phosphatase conjugate (Thermo scientific, Rockford, IL) with 5 mg/ml unbiotinylated hIgG in blocking buffer (PBS+0.05% Tween 20+1% BSA) was used as a secondary reagent. The wells were incubated with the secondary reagent for 1 hour at 4°C, washed again three times and incubated with 100 µl of p-nitrophenyl phosphate substrate (Sigma Aldrich, St. Louis, MO). The absorbance at 405 nm was read in a microplate reader. Triplicate wells coated with cysteine-tagged mutants were used for each concentration of hIgG and the controls. The control wells not incubated with the hIgG were used for background subtraction. All absorbance values were normalized to the absorbance from the wells dissociated with a buffer at pH 7.4.

## Results and Discussion

### Isolation of hFc-binding Proteins from a Library of Sso7d Mutants

We isolated binding proteins from a yeast display library of ∼10^8^ Sso7d mutants using magnetic selection and FACS. A stringent negative selection step was included to eliminate binders to the other non-human immunoglobulins, chicken IgY (cIgY), mouse IgG (mIgG) and rabbit IgG (rIgG). After four rounds of FACS, a pool of mutants with the highest binding affinity for hFc was isolated and individual clones were sequenced. Three distinct hFc-binding Sso7d clones were obtained (Table 1). Our preliminary analysis showed that the clone Sso7d-hFc had the highest binding affinity for hFc (data not shown), and was chosen for further analysis.

**Table 1:** Sequences of hFc-binding Sso7d mutants.

	20…….30…….40…
**wt Sso7d**	**KK**V**W**R**V**GK**M**I**S**F**T**YDLGGGK**T**G**R**G**A**
**hFc-1(4)** a	**SI**V**P**R**S**GK**Y**I**H**F**I**YDLGGGK**T**G**R**G**N**
**hFc-2(4)** a	**CV**V**R**R**F**GK**V**I**S**F**D**YDLGGGK**S**G**R**G**C**
**Sso7d-hFc** b	**YL**V**S**R**I**GK**R**I**L**F**M**YDLGGGK**Y**G**I**G**R**
**Sso7d-his-hFc** b	**YL**V**S**R**I**GK**R**I**L**F**M**YDLGGGK**H**G**I**G**R**

The corresponding sequence for the wild type protein is shown as a reference. Mutated residues are depicted in boldface type and underlined. The sequence for Sso7d-his-hFc (Y40H) is also shown.

a Number in parentheses indicates the number of identical DNA sequences obtained.

b These proteins were chosen for further analysis.

### Sso7d-hFc Binding to hFc does not Exhibit pH Sensitivity

**Figure 1:**
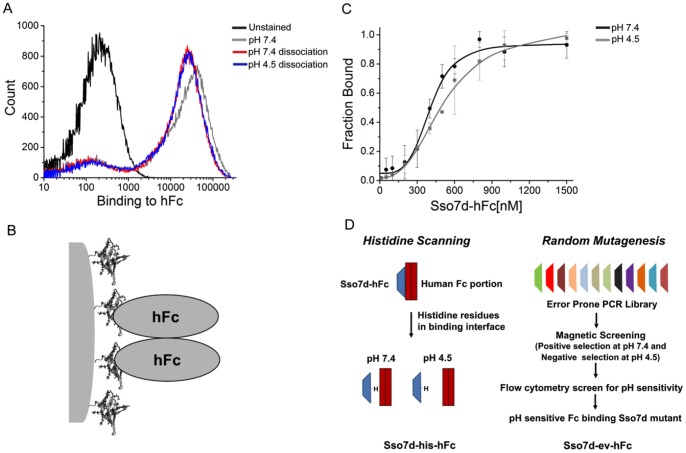
Characterization of pH sensitivity for Sso7d-hFc and strategies for engineering pH sensitivity. (**A**) End-point assay to determine pH sensitivity for Sso7d-hFc. Yeast cells displaying Sso7d-hFc were incubated with 100 nM hFc-biotin and the yeast-hFc complexes were dissociated in buffers at pH 7.4 and pH 4.5. Undissociated hFc remaining on yeast surface was detected using strep-PE. A cell sample where no dissociation step was carried out after hFc labeling at pH 7.4, and unstained cells were used as controls. (**B**) Dimeric hFc may form a multivalent association with cell surface displayed Sso7d-hFc. (**C**) ELISA for determination of the apparent K_D_ of binding between hFc and Sso7d-hFc, at pH 7.4 and pH 4.5. hIgG (1 µg/ml) was immobilized on a microtiter plate and incubated with twelve different concentrations of soluble Sso7d-hFc. hIgG-bound Sso7d-hFc was detected using an anti-his-alkaline phosphatase conjugated antibody, and p-nitrophenyl phosphate as the substrate. Error bars indicate the standard deviation of absorbance measurements at 405 nm. (**D**) Two different strategies used for engineering pH sensitive binding proteins are shown. The first strategy involves mutation of amino acid residues in the binding interface to histidine. The second strategy involves screening of pH sensitive binders from a library generated by random mutagenesis of hFc binders.

We tested the pH sensitivity of the binding interaction between hFc and Sso7d-hFc using a flow cytometry based “end-point” assay. Briefly, yeast cells displaying Sso7d-hFc were labeled with hFc, and complexes were allowed to dissociate for 30 min in a buffer at pH 7.4 or at pH 4.5. Subsequently, the fluorescence signal corresponding to cell-surface bound hFc was measured in each case. This assay bears crude resemblance to chromatographic capture of hFc by immobilized Sso7d-hFc and subsequent elution by buffer at lower pH. As seen in Figure 1A, the fluorescence signal is essentially identical in case of dissociation at pH 7.4 and at pH 4.5. A decrease in pH does not increase the dissociation of hFc from the surface of yeast cells displaying Sso7d-hFc. Thus, Sso7d-hFc binding to hFc does not exhibit pH sensitivity under conditions of the end-point assay. However, it is important to note that binding of the dimeric hFc to cell-surface Sso7d-hFc likely results in a multivalent (2∶2) interaction (Figure 1B). The avidity of this interaction can slow down the rate of dissociation of hFc from the cell surface. Therefore, to eliminate artifacts due to the avidity effect, we measured the apparent equilibrium dissociation constant (K_D_) of the binding interaction between hFc and Sso7d-hFc at pH 7.4 and pH 4.5, using recombinant Sso7d-hFc in an ELISA. Notably, the interaction between immobilized hFc and soluble Sso7d-hFc is not multivalent in the ELISA. As shown in Figure 1C and Table 2, the binding affinity of Sso7d-hFc for hFc at pH 7.4 and pH 4.5 is similar. Taken together, these results confirm that Sso7d-hFc binding to hFc does not exhibit pH sensitivity.

**Table 2:** Apparent equilibrium dissociation constants (K_D_) for the Sso7d mutants.

Sso7d mutant	K_D_ (pH 7.4) (nM)	K_D_ (pH 4.5) (nM)
**Sso7d-hFc**	400 (366–437)	506 (439–628)
**Sso7d-his-hFc**	1647 (1337–3000)	N.D.^a^
**Sso7d-ev-hFc**	5280 (4500–6700)	N.D.^a^

For each mutant, data from at least two independent experiments were fit globally to a four parameter logistic model using non-linear least squares regression for estimation of K_D._ The corresponding 68% confidence intervals are shown in parentheses.

a Not determined. The binding isotherm at pH 4.5 did not reach saturation even with 20–30 µM concentration of Sso7d-his-hFc and Sso7d-ev-hFc; consequently, an apparent K_D_ for the binding interaction at pH 4.5 could not be calculated.

We used two different strategies to engineer pH sensitive hFc binders. First, we used a histidine scanning strategy wherein histidine residues are introduced in the binding interface to engineer pH sensitivity of binding. The second strategy involved selecting pH sensitive binders from a library of Sso7d mutants that was generated through random mutagenesis of a pool of hFc binders. Figure 1D shows an overview of these approaches; our results are described in the following sections.

### Generation of pH Sensitive hFc Binders by Histidine Scanning

**Figure 2:**
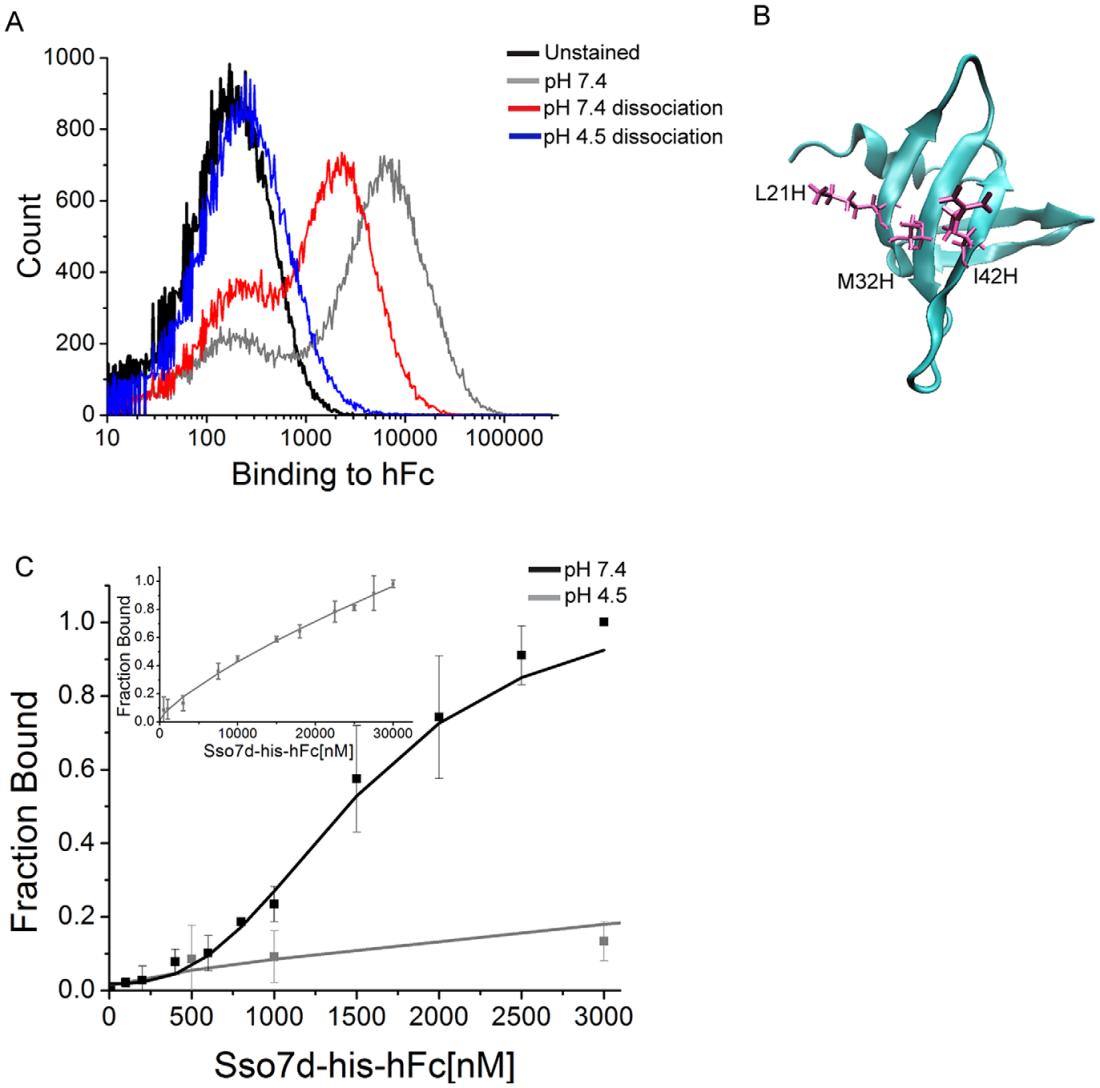
Characterization of pH sensitivity for Sso7d-his-hFc. (**A**) End-point assay to determine pH sensitivity for Sso7d-his-hFc. Yeast cells displaying Sso7d-his-hFc were incubated with 100 nM hFc-biotin and the yeast-hFc complexes were dissociated in buffers at pH 7.4 and pH 4.5. Undissociated hFc remaining on yeast surface was detected using strep-PE. A cell sample where no dissociation step was carried out after hFc labeling at pH 7.4, and unstained cells were used as controls. (**B**) Histidine scanning analysis of Sso7d-hFc. Three out of ten histidine substitutions (L21H, M32H and I42H) lead to complete loss of binding to hFc, and therefore are involved in binding to hFc. Structure of the Sso7d scaffold with these residues in licorice representation is shown. This image was generated using Visual Molecular Dynamics (VMD) software. (**C**) ELISA for determination of the apparent K_D_ of binding between hFc and Sso7d-his-hFc, at pH 7.4 and pH 4.5. hIgG (2 µg/ml) was immobilized on a microtiter plate and incubated with twelve different concentrations of soluble Sso7d-his-hFc. hIgG-bound Sso7d-his-hFc was detected using an anti-his-alkaline phosphatase conjugated antibody, and p-nitrophenyl phosphate as the substrate. Error bars indicate the standard deviation of absorbance measurements at 405 nm. The inset shows binding curve at pH 4.5 over a wider range of Sso7d-his-hFc concentration.

Histidine scanning has been extensively used to introduce pH sensitivity of binding in the context of therapeutic antibody engineering and cytokine design. A hallmark of this approach is the introduction of one or more histidine residues in the binding interface of a protein or an engineered mutant. A crystal structure or molecular modeling may help in predicting residues to be mutated [5]. Here, we performed histidine scanning mutagenesis of Sso7d-hFc by systematically replacing ten residues in the putative binding interface with histidine, one at a time, and testing the pH sensitivity of binding using the end-point assay described in the previous section. Note that Sso7d-hFc was obtained by mutagenesis of Sso7d at these ten positions. Results for the end-point assay for all mutants can be found in Figure S1. One of the mutants, Y40H (denoted as Sso7d-his-hFc here on) showed significant pH sensitivity of binding (Figure 2A) and was selected for further analysis. Interestingly, three out of the ten mutations – L21H, M32H and I42H – resulted in loss of binding to hFc, suggesting that L21, M32 and I42 are likely involved in the binding of Sso7d-hFc to hFc (Figure 2B). We determined the apparent K_D_ of the interaction between hFc and recombinant Sso7d-his-hFc by ELISA, at pH 7.4 and pH 4.5. Our results are presented in Table 2 and Figure 2C. The Y40H substitution caused a decrease in binding affinity of Sso7d-hFc for hFc. Nevertheless, the binding affinity of Sso7d-his-hFc for hFc is significantly higher at pH 7.4 than at pH 4.5. The binding isotherm at pH 4.5 did not reach saturation even with 30 µM concentration of Sso7d-his-hFc; consequently, an apparent K_D_ for the binding interaction at pH 4.5 could not be calculated. Thus, our results show that a pH sensitive hFc binder could be obtained by systematic histidine scanning mutagenesis of Sso7d-hFc.

### Generation of pH Sensitive hFc Binders by Random Mutagenesis

Previous studies [3], [4], [6] as well as our results in this study show that histidine scanning is indeed an effective approach to engineer pH sensitivity of binding. However, a potential limitation of this approach is that the sequence space of mutant proteins searched is restricted to histidine substitutions. Additionally, our histidine scanning study introduced mutations only at surface residues. Mutations in the core of the protein as well as non-histidine residues may contribute to pH sensitivity of binding [7]. Therefore, we also sought to generate pH sensitive binders through random mutagenesis of hFc-binding Sso7d mutants. Specifically, our goal was to select mutants that have a higher binding affinity for hFc at pH 7.4 than at pH 4.5.

Conceptually, a pH sensitive mutant can be obtained by selectively introducing higher binding affinity at pH 7.4 in a low affinity binder, or conversely lower affinity at pH 4.5. Therefore, to maximize the likelihood of isolating a pH sensitive binder, DNA from two pools of hFc binders obtained during the isolation of Sso7d-hFc – a low affinity pool obtained after the magnetic sort and a higher affinity pool obtained after two rounds of FACS – was used as the starting point for construction of another yeast display library of ∼ 10^7^ mutants. Error-prone PCR in the presence of nucleotide analogues [11]–[13] was used to introduce random mutations in Sso7d mutants with hFc-binding activity. This library was screened using magnetic selection and FACS to isolate pH sensitive hFc binders.

**Figure 3:**
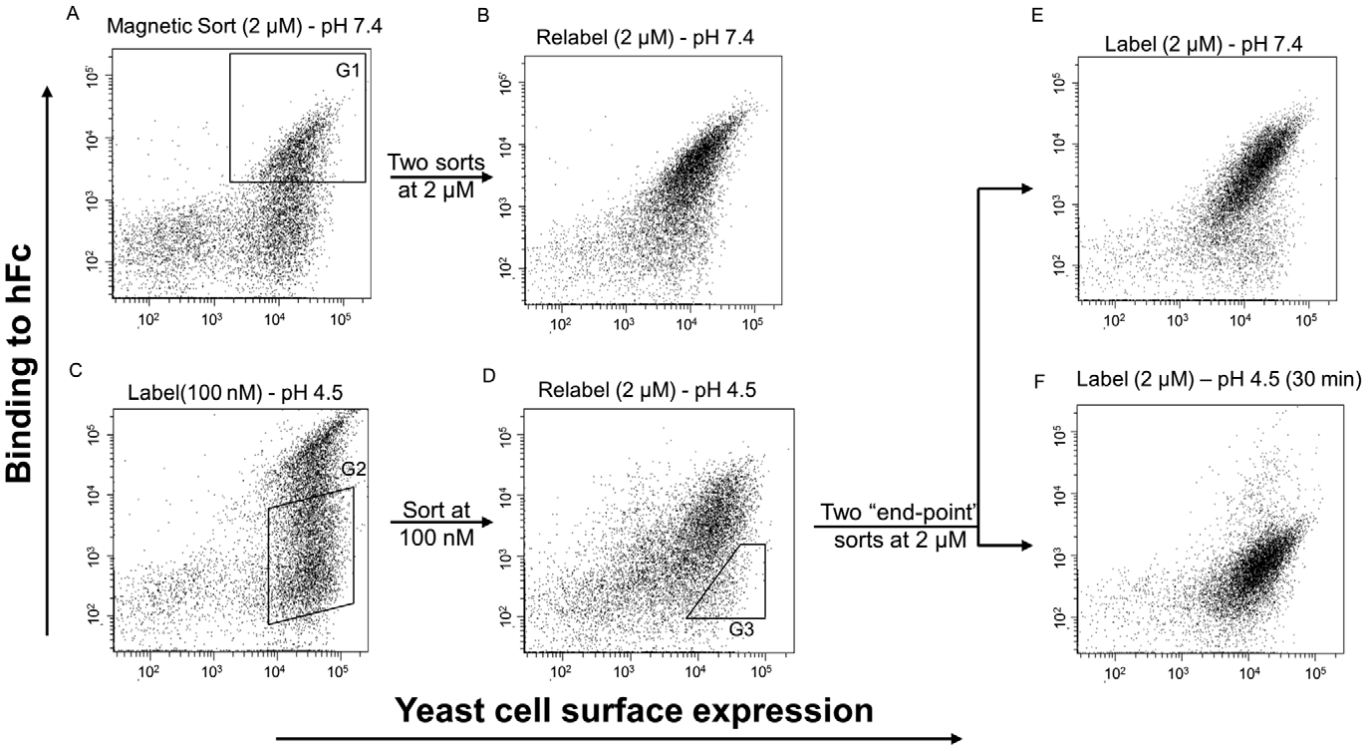
Screening strategy for isolating pH sensitive binding proteins using fluorescence activated cell sorting. The library obtained by random mutagenesis of a pool of hFc binders was screened using magnetic selection and flow cytometry. (**A**) The mutant population after magnetic selection was labeled with 2 µM hFc-biotin at pH 7.4 and sorted twice for isolating hFc-binding mutants with higher binding affinity (gate G1). (**B**) shows the population after two consecutive sorts, labeled at pH 7.4 and 2 µM hFc-biotin. (**C**) The population from (B) was labeled with 100 nM hFc-biotin at pH 4.5 and further sorted to isolate mutants with loss of binding to hFc-biotin as shown (gate G2). (**D**) shows the population from (C) labeled at 2 µM hFc-biotin and pH 4.5. This population was sorted twice using an end-point sort strategy wherein Sso7d mutants were labeled at 2 µM hFc-biotin and pH 7.4, and mutants that lose binding to hFc-biotin after a 30 min dissociation at pH 4.5 are selected (gate G3). All the mutants in the final pool bound hFc-biotin at pH 7.4 (**E**) and lost binding to hFc-biotin at pH 4.5 (**F**). In all sorts, cells were also simultaneously labeled with an antibody against the c-myc epitope tag.

**Figure 4:**
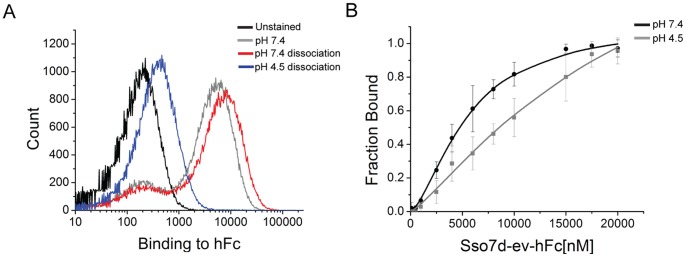
Characterization of pH sensitivity for Sso7d-ev-hFc. (**A**) End-point assay to determine pH sensitivity for Sso7d-ev-hFc. Yeast cells displaying Sso7d-his-hFc were incubated with 2 µM hFc-biotin and the yeast-hFc complexes were dissociated in buffers at pH 7.4 and pH 4.5. Undissociated hFc remaining on yeast surface was detected using strep-PE. A cell sample where no dissociation step was carried out after hFc labeling at pH 7.4, and unstained cells were used as controls. (**C**) ELISA for determination of the apparent K_D_ of binding between hFc and Sso7d-ev-hFc, at pH 7.4 and pH 4.5. hIgG (2 µg/ml) was immobilized on a microtiter plate and incubated with twelve different concentrations of soluble Sso7d-ev-hFc. hIgG-bound Sso7d-ev-hFc was detected using an anti-his-alkaline phosphatase conjugated antibody, and p-nitrophenyl phosphate as the substrate. Error bars indicate the standard deviation of absorbance measurements at 405 nm.

First, a negative selection was employed to eliminate yeast cells that bound to the biotin-binder magnetic beads. Subsequently, yeast cells that bound hFc-coated magnetic beads at pH 7.4 were isolated using a magnet. After two washes at pH 7.4, the yeast-bead complexes were incubated with shaking in pH 4.5. Dissociated cells were collected and expanded in SDCAA media. This pool of cells was further sorted by FACS (Figure 3). Two sorts were performed at 2 µM hFc labeling and pH 7.4 (Figure 3A) to select for all hFc-binding Sso7d mutants at pH 7.4. This hFc-binding population (Figure 3B) was then labeled at a lower concentration (100 nM) and pH 4.5 to select for clones that lose binding at pH 4.5 (Figure 3C). Following this step, two end-point sorts were performed as follows. In each sort, yeast cells displaying Sso7d mutants were incubated with 2 µM hFc at pH 7.4 and allowed to reach equilibrium. These yeast-hFc complexes were then dissociated in pH 4.5 for 30 min with shaking, and cells were sorted for loss of binding (Figure 3D). After two end-point sorts, a pure population was obtained where the mutants bound hFc at pH 7.4 and lost binding at pH 4.5 (Figure 3E and 3F). Ten individual clones were picked from this final population for analysis by the end-point assay described earlier (Figure S2). The sequences of isolated clones are shown in Table 3. Sso7d-ev-hFc was selected as the best binding clone based on its ability to have the maximum change in binding between pH 7.4 and pH 4.5 in the end-point assay (Figure 4A), and used in further analysis. As in case of Sso7d-hFc and Sso7d-his-hFc, we measured the apparent K_D_ of the interaction between hFc and recombinant Sso7d-ev-hFc by ELISA, at pH 7.4 and pH 4.5 (Table 2 and Figure 4B). As with Sso7d-his-hFc, the binding isotherm at pH 4.5 did not reach saturation even with 20 µM concentration of Sso7d-ev-hFc. Therefore, an apparent K_D_ for the binding interaction at pH 4.5 could not be calculated. Nevertheless, the binding affinity of Sso7d-ev-hFc for hFc is significantly greater at pH 7.4 than at pH 4.5.

**Table 3:** Sequences of pH-sensitive binding proteins isolated from a library of Sso7d mutants, obtained by random mutagenesis of a pool of hFc binders.

	….20…….30…….40…….50….
**Sso7d-hFc**	DISKI**YL**V**S**R**I**GK**R**I**L**F**M**YDLGGGK**Y**G**I**G**R**VSEKDAPKELL
**hFcev1**	DIS**E**I**YR**V**S**R**R**GK**S**I**A**F**M**YDLGGGK**Y**G**I**G**Y**VSEKDAPKELL
**hFcev2(2)** a	DISKI**FF**V**R**R**ID**K**L**I**A**F**S**YDLGGGK**Y**G**L**G**F**VSEKDAPKELL
**hFcev4**	DISKI**RL**V**S**R**N**G**RR**I**S**F**M**YDLGGGK**H**G**I**G**Q**VSE**R**DAPKELL
**hFcev6**	DISKI**RL**V**S**R**Q**GK**I**I**K**F**T**YDLGGG**EL**G**M**G**R**VSEKDAPKELL
**Sso7d-ev-hFc** b	DISKI**YR**V**F**R**S**GK**T**I**F**F**R**YDLGGG**EL**G**V**G**I**VSEKDAPKELL
**hFcev9**	DISKI**RL**V**A**R**T**GK**I**I**R**F**Q**YDLGGGK**Y**G**L**G**R**VSEKDAPKELL
**hFcev10**	DISKI**YR**V**S**R**R**G**ES**I**A**F**M**YDLGGGK**Y**G**I**G**Y**VSE**E**DAP**E**ELL

The sequence of Sso7d-hFc is shown as a reference. Mutated residues are depicted in boldface type and underlined.

a Number in parenthesis indicates the number of identical DNA sequences obtained.

b This protein was chosen for further analysis.

Strikingly, the apparent K_D_ at pH 7.4 for Sso7d-ev-hFc is much higher than the corresponding values for Sso7d-hFc and Sso7d-his-hFc. This is a clear illustration of the axiom “you get what you screen for” that applies to directed evolution in general [16]. We selected yeast cells forming cell-surface complexes with hFc at pH 7.4, such that the complexes dissociate upon incubation at pH 4.5– but not at pH 7.4– for 30 min. Due to avidity of interaction between the dimeric hFc and cell-surface displayed mutant proteins, the rate of dissociation of cell-surface complexes can slow down greatly. Consequently, even a mutant with low binding affinity can form cell-surface complexes that remain undissociated after incubation for 30 minutes at pH 7.4. Thus, even though we did not explicitly search for a low affinity hFc binder, Sso7d-ev-hFc satisfied all the selection criteria and was isolated in our screen.

### Biophysical Characterization of Sso7d-hFc, Sso7d-his-hFc and Sso7d-ev-hFc

**Figure 5:**
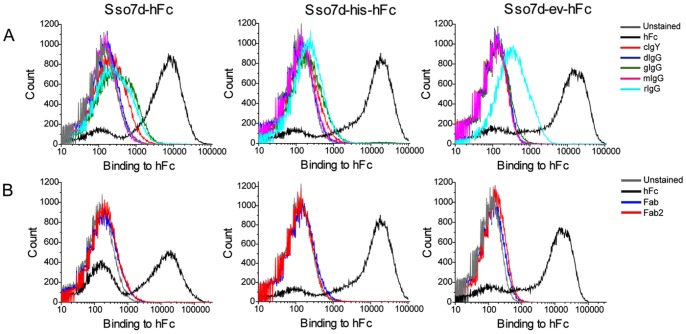
Specificity of Sso7d-hFc, Sso7d-his-hFc and Sso7d-ev-hFc. (**A**) Yeast displayed Sso7d-hFc, Sso7d-his-hFc and Sso7d-ev-hFc were labeled with 1 µM hFc or 1 µM closely related non-human immunoglobulins, chicken IgY (cIgY), donkey IgG (dIgG), goat IgG (gIgG), mouse IgG (mIgG) and rabbit IgG (rIgG). Specific binding to hFc was observed. (**B**) Mutants were also labeled with hIgG fragments hFc, Fab and Fab2. Binding to Fab and Fab2 fragments was not observed.

We evaluated the binding specificity of Sso7d-hFc, Sso7d-his-hFc and Sso7d-ev-hFc to other closely related IgGs and Fab fragments through a flow cytometry assay. Yeast cell-surface displayed hFc binders were labeled with 1 µM of hFc, mIgG, cIgY, rIgG, goat IgG (gIgG), donkey IgG (dIgG), Fab and Fab2. As shown in Figure 5A, Sso7d-hFc, Sso7d-his-hFc and Sso7d-ev-hFc bind specifically to hFc. Sso7d-ev-hFc showed minimal binding to rabbit IgG. Also, all three mutants do not bind the Fab and Fab2 fragments (Figure 5B).

**Figure 6:**
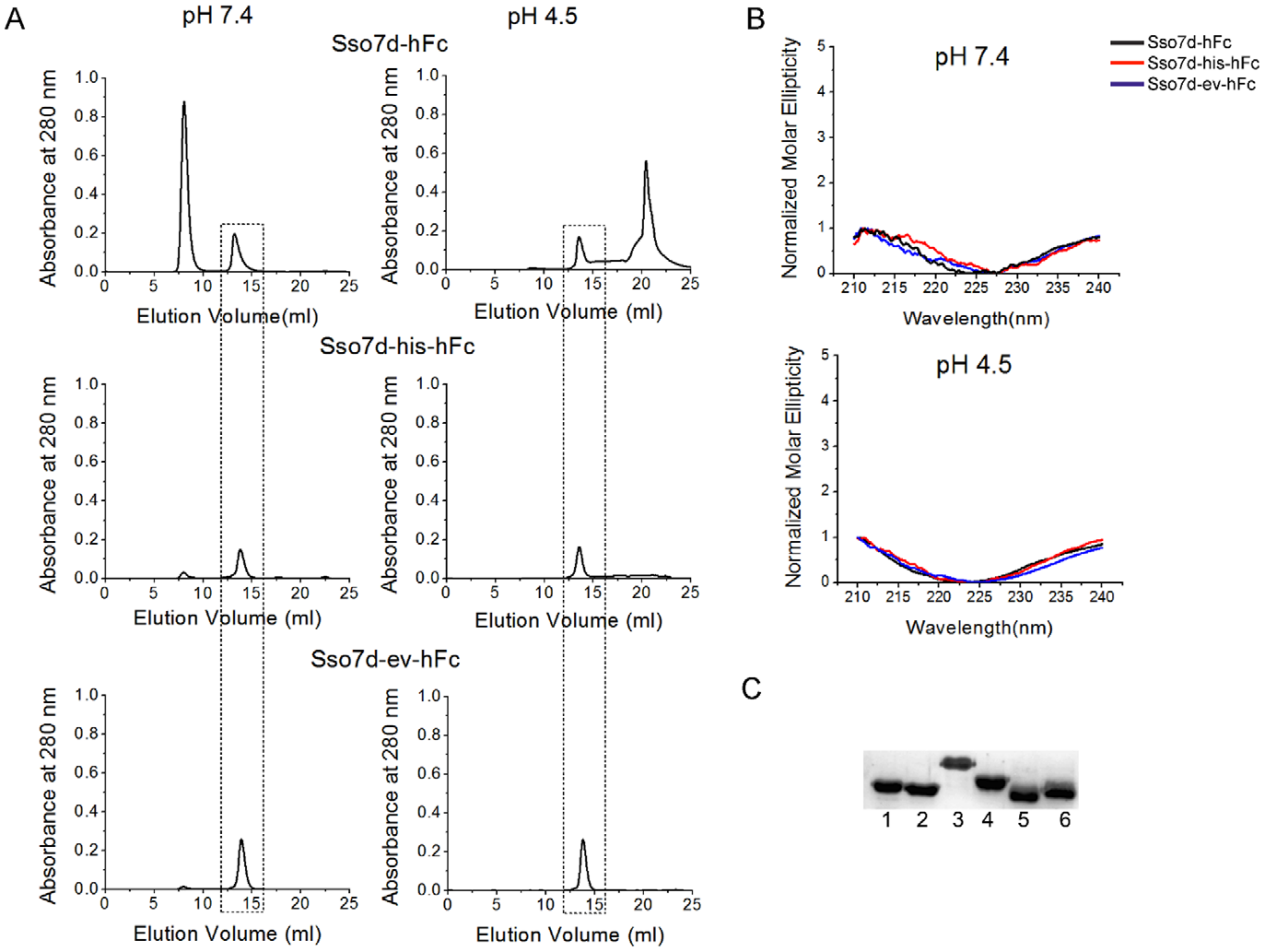
Biophysical characterization of hFc binding Sso7d mutants and western blotting analysis. (**A**) Size exclusion chromatography of Sso7d mutants purified by immobilized metal affinity chromatography (IMAC). The dashed box indicates elution peak for Sso7d mutants. Mutants were loaded on the column at a concentration of 2 mg/ml. Molecular weight estimates based on the retention time of Sso7d mutants in the column are consistent with the mutants being present in monomeric form. The other peak corresponds to a minor impurity with higher molar absorptivity than the Sso7d mutants (see Figure S3; SDS-PAGE analysis of fractions corresponding to the other peak do not show any detectable protein). (**B**) Circular dichroism spectra for Sso7d-hFc, Sso7d-his-hFc and Sso7d-ev-hFc at pH 7.4 and pH 4.5. The spectra at both pH values is essentially the same confirming that there is no change in secondary structure when the pH is lowered from 7.4 to 4.5 (**C**) Sso7d-hFc recognizes all four hIgG isotypes as well as the deglycosylated form of hIgG, when used as a primary reagent for detection in western blotting analysis. Lane 1: hIgG_1_, lane 2: hIgG_2_, lane 3: hIgG_3_, lane 4: hIgG_4_, lane 5: hIgG digested with PNGase F, lane 6: undigested hIgG (control). Similar results were observed with Sso7d-his-hFc and Sso7d-ev-hFc (data not shown).

All three Sso7d mutants could be expressed recombinantly in the *E. coli* cytoplasm with a C-terminal 6x-his tag. Estimated yields of purified proteins were 40–50 mg per liter of unoptimized shake flask culture. Molecular weights estimates, obtained using analytical size exclusion chromatography, suggest that all recombinant proteins are monomeric at a concentration of 2 mg/ml (Figure 6A). We also conducted CD measurements at pH 7.4 and pH 4.5 to confirm that the pH sensitivity of Sso7d-his-hFc and Sso7d-ev-hFc was not due to a change in secondary structure. As seen from the normalized CD spectra in Figure 6B, all mutants show similar secondary structure at the two different pH values. Notably, the normalized CD spectra of the Sso7d mutants are also similar to that of wild-type Sso7d and other Sso7d-based mutant binding proteins at these values of pH [8]. We have previously shown that wild-type Sso7d and several Sso7d mutants retain their secondary structure over a wide range of pH (pH 0.33–12.5). Finally, we carried out western blotting analysis to evaluate binding of Sso7d-hFc, to the hIgG subclasses hIgG_1_, hIgG_2_, hIgG_3_ and hIgG_4_. A biotinylated form of recombinant Sso7d-hFc in conjunction with an anti-biotin antibody conjugated to horse radish peroxidase (HRP). As shown in Figure 6C, Sso7d-hFc recognizes all four hIgG subclasses in the context of western blotting. Additionally, Sso7d also recognizes deglycosylated hIgG obtained by treatment of hIgG with PNGase. Similar results were obtained with Sso7d-his-hFc and Sso7d-ev-hFc (data not shown).

### Epitope Mapping Using Yeast Surface Display

**Figure 7:**
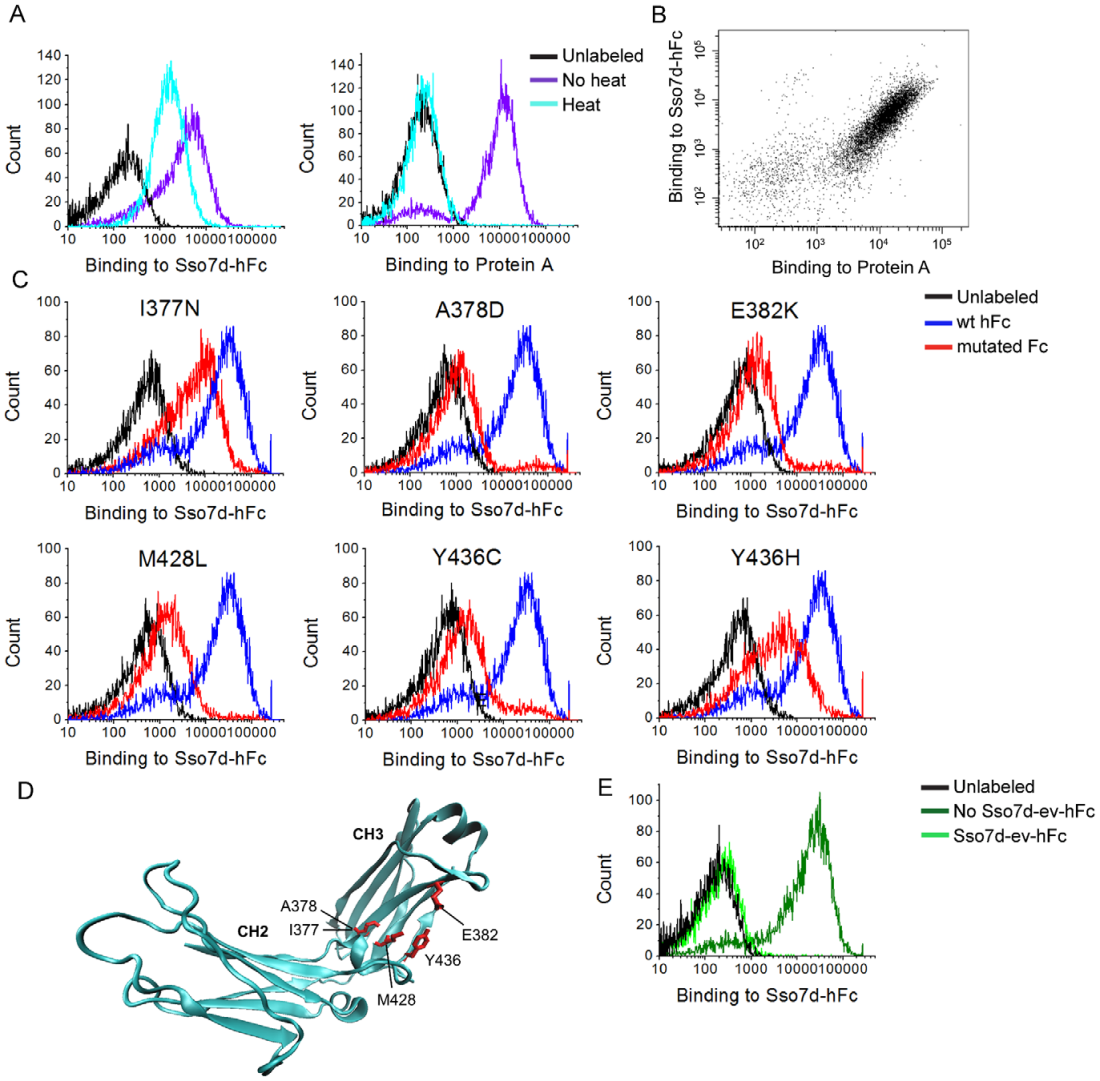
Epitope mapping of hFc binding Sso7d mutants. (**A**) Yeast surface displayed hFc was subjected to thermal denaturation at 99°C and subsequently detected using Sso7d-hFc or Protein A. Binding to Protein A is completely abolished, whereas binding to Sso7d-hFc is only slightly reduced. (**B**) Protein A and Sso7d-hFc can simultaneously detect the yeast-displayed hFc, confirming that they have distinct binding epitopes on hFc. (**C**) Individual hFc mutants from the final epitope mapping sorts are shown. These mutants lead to loss of binding of Sso7d-hFc to yeast displayed hFc (also see Table 4). (**D**) The mutations in (C) are mapped on the crystal structure of hFc. (**E**) Yeast surface displayed hFc was incubated with biotinylated Sso7d-hFc in the presence or absence of a high concentration of (unbiotinylated) Sso7d-ev-hFc. Cell surface bound hFc was detected using streptavidin-phycoerythrin (strep-PE). Sso7d-ev-hFc competes off the bound Sso7d-hFc confirming that Sso7d-ev-hFc binds an epitope that overlaps at least partially with that of Sso7d-hFc.

We expressed hFc as a cell-surface fusion using yeast display. Immunofluorescent detection with Protein A confirmed that hFc expressed on the yeast cell surface was functional and correctly folded (Figure 7A, 7B). Interestingly, binding of yeast-displayed hFc to Protein A is completely abolished after heat treatment at 99°C for 30 min. By contrast, binding to Sso7d-hFc is partially retained (Figure 7A). These findings are consistent with results from western blotting where denatured IgG could be detected by Sso7d-hFc. Additionally, Sso7d-hFc and Protein A could simultaneously bind to yeast-displayed hFc (Figure 7B), confirming that these proteins have distinct binding epitopes on hFc.

We further used yeast surface display to determine the binding epitope on hFc that interacts with Sso7d-hFc. Simplistically, point mutations in hFc that result in loss of binding to Sso7d-hFc, indicating a residue critical for the binding interaction, were identified. This approach has been effectively used for epitope mapping, including discontinuous and conformational epitopes [17], [18]. Notably, a key advantage of using yeast display for epitope mapping is that soluble expression of mutant proteins is not required. A library of 6×10^6^ hFc mutants was generated by random mutagenesis, using error prone PCR with a low rate of mutation. DNA Sequencing analysis of 23 library clones showed 14 wild type hFc clones (61%), 5 mutants with single amino acid substitutions (22%) and 4 clones with multiple amino acid substitutions or deletions (17%). Crude estimates based on these numbers suggest that the total number of clones in the library with single amino acid substitutions (∼ 1.3×10^6^) far exceeds the theoretical diversity of hFc mutants with single amino acid mutations (4.6×10^3^). However, it must be noted that mutation of a given residue to every other amino acid cannot be achieved by single nucleotide changes alone. Therefore the library does not oversample every possible single amino acid mutation at each residue. Nonetheless, the oversampling of all single amino acid mutations is not required to gain insight into the binding epitope.

We selected hFc mutants that exhibit loss of binding to Sso7d-hFc using FACS. Certain mutations may cause misfolding of hFc. Therefore, we used Protein A as a positive control to probe for proper folding of the hFc mutants. Mutants exhibiting loss of binding to Sso7d-hFc, but not Protein A were selected. These mutants putatively contain mutations at contact residues responsible for binding to hFc. After 3 rounds of FACS, we sequenced 50 different clones from the pool of selected mutants and those with single point mutations were identified (Table 4). Among these, the hFc mutants A378D, E382K, M428L and Y436C show loss of binding to hFc; I377N and Y436H showed partial loss of binding (Figure 7C, Table 4). These amino acid positions constitute at least a subset of residues that are likely to be involved in the binding interaction of Sso7d-hFc with hFc; they are mapped on the crystal structure of hFc in Figure 7D. All mutations lie in the CH3 region of hFc on a β-sheet. Interestingly, the amino acid sequence of hIgG_1–4_ is highly conserved in this region, consistent with our results from western blotting. Also, as discussed earlier, our histidine scanning experiments show that mutations that L21, M32 and I42 in Sso7d-hFc are likely involved in the binding interaction of Sso7d-hFc with hFc (Figure 2B).

**Table 4:** Mutations in hFc leading to loss of binding to Sso7d-hFc.

hFc mutant	Binding to Sso7d-hFc	Binding to Protein A
**hFc-wild type**	++	++
**I377N**	+	++
**A378D**	–	++
**E382K**	–	++
**M428L,I,V,T**	–	++
**Y436C**	–	++
**Y436H**	+	++

Amino acid changes in mutants with single amino acid substitutions that retain binding to Protein A but lose binding to Sso7d-hFc were selected as critical residues involved in the binding epitope of Sso7d-hFc on hFc.

(++) indicates wild type level binding;

(+) indicates binding somewhat reduced and.

(–) indicates loss of binding.

Sso7d-his-hFc differs from Sso7d-hFc by only one histidine residue. Therefore, it is reasonable to assume that Sso7d-his-hFc binds the same epitope on hFc as Sso7d-hFc. Due to the low binding affinity of Sso7d-ev-hFc, epitope mapping using yeast display was not feasible. Binding of Sso7d-ev-hFc to yeast-displayed hFc cannot be detected by flow cytometry. This is likely due to the dissociation hFc-Sso7d-ev-hFc complexes during the wash steps in the absence of the avidity effect, as is the case when hFc is displayed on the surface of yeast. However, a competition experiment showed that Sso7d-ev-hFc binds an epitope that overlaps – at least partially – with that of Sso7d-hFc. Binding of yeast-displayed hFc to soluble Sso7d-hFc (2 µM) was completely abolished in the presence of an excess of Sso7d-ev-hFc (200 µM) (Figure 7E). Thus, in summary, we have identified a region on hFc that putatively mediates the binding interaction with all three hFc-binding Sso7d mutants evaluated in this study. However, further structural data, such as a crystal structure of the hFc-protein complex, is needed to elucidate the exact mechanism of pH sensitivity for Sso7d-his-hFc and Sso7d-ev-hFc.

### pH Sensitivity of hFc Dissociation from a Surface is Influenced by Avidity as well as Surface Density of Immobilized Sso7d Mutants

Our end-point assays for assessing pH sensitivity crudely resemble chromatographic capture of hFc by immobilized Sso7d-hFc and subsequent elution by a buffer at lower pH. A low pH buffer (pH 2–3) is often used for elution of hIgG from a capture surface in chromatography. Low elution pH may cause denaturation or aggregation of the IgG product and necessitate further refolding steps, making the overall process expensive and complicated [19]. Therefore, binders that enable elution of hIgG at a relatively higher, milder pH are desirable. A previous study has reported the generation of a pH sensitive variant of protein G variant that can release IgG from a capture surface at pH of 4.3 [20]. Additionally, variants of Protein A with lower binding affinity for IgG can be used to elute IgG at higher pH [21], [22]. Both Sso7d-his-hFc and Sso7d-ev-hFc exhibit pH sensitivity of hFc-binding, and have a lower binding affinity for hFc than Sso7d-hFc. Therefore, we examined if Sso7d-his-hFc and Sso7d-ev-hFc, can elute hIgG at a pH higher than Sso7d-hFc, when immobilized on a surface.

**Figure 8:**
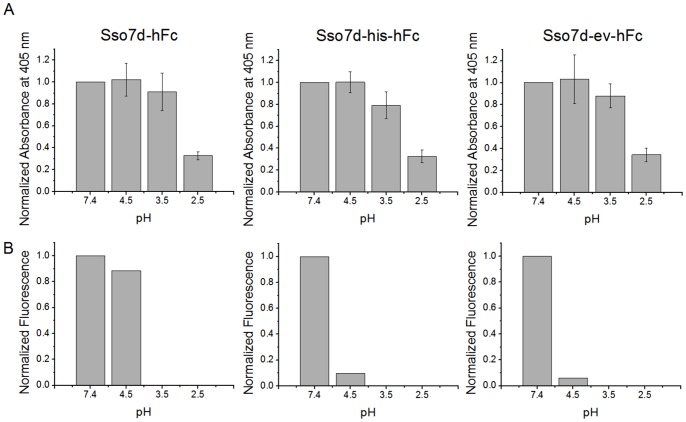
pH sensitivity hFc binding to Sso7d mutants immobilized on a surface. (**A**) Sso7d-hFc, Sso7d-his-hFc and Sso7d-ev-hFc were recombinantly expressed with a C-terminal cysteine. 2 µg/ml of each mutant was chemically conjugated to wells of a maleimide-activated microtiter plate and incubated with 6 µM of hIgG-biotin. After a wash step, wells were incubated with buffers of pH 7.4, 4.5, 3.5 and 2.5 for 30 min with shaking, and the hIgG-biotin remaining undissociated was detected with alkaline phosphatase conjugated streptavidin and p-nitrophenyl-phosphate; absorbance measurements at 405 nm on a plate reader were obtained. Background-subtracted absorbance values were normalized by the absorbance value corresponding to dissociation in buffer at pH 7.4, and are reported. Error bars indicate standard deviation of measurements from six different wells. All three mutants show similar dissociation behavior in buffers at different pH. (**B**) Data from end-point assays for pH sensitivity of Sso7d mutants (see Figures 1A, 2A, 4A) are analyzed for comparison with (A). As in (A), Sso7d mutants immobilized on the surface of yeast are incubated with hFc-biotin and subsequently, the surface-bound hFc complexes are allowed to dissociate for 30 min in a buffer at pH 7.4 or pH 4.5; experiments with dissociation at pH 3.5 and pH 2.5 were not conducted. The fluorescence due to surface-bound hFc remaining undissociated was measured by flow cytometry, following labeling with strep-PE. Mean fluorescence values, normalized to the value for dissociation with pH 7.4 buffer, are plotted; data shown is the average from two separate experiments. A significantly greater fraction of hFc remains bound to Sso7d-hFc immobilized on the yeast cell surface.

Sso7d mutants containing a C-terminal cysteine were conjugated to wells of a microtiter plate containing a reactive maleimide group, to prepare test capture surfaces with immobilized hFc binders. These surfaces were incubated with biotinylated hIgG, and following a wash step, buffers with varying pH (pH 7.4, 4.5, 3.5 and 2.5) were used to dissociate the captured IgG. The bound hIgG remaining undissociated was determined using a colorimetric assay. Our results are shown in Figure 8A. Strikingly, all three mutants show similar dissociation behavior over the pH range tested, despite the pH sensitivity of hFc-binding in case of Sso7d-his-hFc and Sso7d-ev-hFc (Table 2). By contrast, in a similar assay discussed earlier (Figures 1A, 2A and 4A), a significantly greater fraction of hFc remains bound to Sso7d-hFc immobilized on the yeast cell surface (Figure 8B). Notably, in both the microtiter well and yeast surface assays, dimeric hFc can form a multivalent interaction with the immobilized hFc binders. However, the surface density of immobilized protein is likely to differ in the two assays. Assuming expression of 5×10^4^–10^5^ copies of a protein on a yeast cell of diameter 5 µm, the average surface density of yeast-displayed protein is 10^–15^–2×10 ^-15^µmol/µm^2^. On the other hand, a monolayer of Sso7d mutant protein immobilized on surface of a microtiter plate well crudely corresponds to a surface density of 3×10^–13^ µmol/µm^2^ (assuming the Sso7d mutants with molecular weight 8.3 kDa as spheres of radius 1.3 nm [23]). These estimates of surface density are average values; clearly, the inhomogeneity of protein distribution on the yeast cell surface or in the microtiter well will affect the local surface density. Nevertheless, our calculations suggest that the surface density of immobilized protein is likely to be significantly lower in case of yeast surface display.

Thus the dissociation behavior of hFc from surfaces functionalized with hFc binders depends on the surface density of immobilized binding proteins. Additionally, the avidity effect slows down dissociation of dimeric hFc from the capture surface. Our results are consistent with chromatography studies on resins with immobilized IgG-binding peptides, where a lower pH is required for elution of IgG when a resin with higher immobilized peptide density is used [24].

### Conclusions

In this study, we have used two different strategies – histidine scanning and random mutagenesis – to generate pH sensitive binding proteins for hFc. We isolated an hFc-binding protein, Sso7d-hFc, through mutagenesis of the hyperthermophilic Sso7d scaffold. Systematic analysis of Sso7d-hFc variants with single histidine substitutions in the binding interface identified the Y40H mutant, Sso7d-his-hFc, as a pH sensitive hFc-binding protein. We also developed a screening strategy to isolate the pH sensitive hFc binder, Sso7d-ev-hFc, from a library of Sso7d mutants, generated by random mutagenesis of a pool of hFc binders. We further used yeast surface display to identify a region in the CH3 domain of hFc as a putative binding epitope for the hFc binders. Notably, the binding epitope lies in region that has high sequence homology in all four hIgG isotypes. Indeed, all hIgG isotypes as well as the deglycosylated form of IgG is recognized by the hFc binders in western blotting assays.

pH sensitive hFc binders are attractive candidates as affinity ligands that elute IgG under milder pH conditions in chromatography applications. However, the pH dependence of dissociation from a capture surface is influenced by surface density of immobilized binding proteins, as well as the avidity effect arising from the multivalent interaction of a multimeric target with the capture surface. Due to these effects, pH sensitivity as measured by K_D_ of a monovalent interaction, or in the context of yeast surface display, is unlikely to directly translate to an equivalent pH sensitivity of dissociation from a capture surface. Further studies are needed to evaluate if the Sso7d mutants identified in this study are indeed useful as affinity ligands for chromatographic applications. If necessary, achieving further increases in pH sensitivity of hFc binding may be explored through one of the following strategies: combining the Y40H mutation in Sso7d-his-hFc with the random mutations identified in this study that lead to pH sensitivity, an additional round of mutagenesis and screening on the pool of pH sensitive mutants obtained by random mutagenesis, or a combination of these two aforementioned approaches.

## Supporting Information

Figure S1 **End-point assay analysis of Sso7d-hFc mutants with single histidine substitutions, to evaluate pH sensitivity.** Yeast cells displaying Sso7d- hFc mutants with single histidine substitutions were incubated with 100 nM hFc-biotin and the yeast-hFc complexes were dissociated in buffers at pH 7.4 and pH 4.5. Undissociated hFc remaining on yeast surface was detected using streptavidin-phycoerythrin (strep-PE). A cell sample where no dissociation step was carried out after hFc labeling at pH 7.4, and unstained cells were used as controls. Sso7d-his-hFc was identified as a pH sensitive hFc-binder and used in further analysis.
(TIF)

Figure S2 **End-point assay analysis of individual clones isolated from a library generated by random mutagenesis of a pool of hFc-binders, to evaluate pH sensitivity.** Yeast cells displaying pH sensitive hFc binders were incubated with 2 µM hFc-biotin and the yeast-hFc complexes were dissociated in buffers at pH 7.4 and pH 4.5. Undissociated hFc remaining on yeast surface was detected using strep-PE. A cell sample where no dissociation step was carried out after hFc labeling at pH 7.4, and unstained cells were used as controls. Sso7d-ev-hFc was identified as the best pH sensitive hFc-binder and used in further analysis.
(TIF)

Figure S3 **SDS PAGE analysis of size exclusion chromatography fractions.** Samples were collected every 1 ml during size exclusion and run on an SDS PAGE Gel as shown. **(A)** Lane 1: Ladder, 2: Sso7d-hFc pH 7.4 (8 ml), 3: Sso7d-hFc pH 7.4 (9 ml), 4: Sso7d-hFc pH 7.4 (10 ml), 5: Sso7d-hFc pH 7.4 (13 ml), 6: Sso7d-hFc pH 7.4 (14 ml), 7: Sso7d-hFc pH 7.4 (15 ml), 8: Sso7d-his-hFc pH 7.4 (8 ml), 9: Sso7d-his-hFc pH 7.4 (9 ml), 10: Sso7d-his-hFc pH 7.4 (10 ml) 11: Sso7d-his-hFc pH 7.4 (13 ml), 12: Sso7d-his-hFc pH 7.4 (14 ml) **(B)** Lane 1: Sso7d-his-hFc pH 7.4 (15 ml), 2: Ladder, 3: Sso7d-ev-hFc pH 7.4 (8 ml), 4: Sso7d-ev-hFc pH 7.4 (9 ml), 5: Sso7d-ev-hFc pH 7.4 (10 ml), 6: Sso7d-ev-hFc pH 7.4 (13 ml), 7: Sso7d-ev-hFc pH 7.4 (14 ml), 8: Sso7d-ev-hFc pH 7.4 (15 ml), 9: Sso7d-hFc pH 4.5 (13 ml), 10: Sso7d-hFc pH 4.5 (14 ml) 11: Sso7d-hFc pH 4.5 (15 ml), 12: Sso7d-hFc pH 4.5 (20 ml) **(C)** Lane 1: Sso7d-hFc pH 4.5 (21 ml), 2: Sso7d-hFc pH 4.5 (22 ml), 3: Ladder, 4: Sso7d-his-hFc pH 4.5 (13 ml), 5: Sso7d-his-hFc pH 4.5 (14 ml), 6: Sso7d-his-hFc pH 4.5 (15 ml), 7: Sso7d-ev-hFc pH 4.5 (13 ml), 8: Sso7d-ev-hFc pH 4.5 (14 ml), 9: Sso7d-ev-hFc pH 4.5 (15 ml). The volume in brackets corresponds to the volume of elution (the x-axis in Figure 6A). Sso7d-mutants elute at the volume corresponding to the monomeric protein and other fractions do not show any protein confirming that these mutants are monomeric and the extraneous peaks in the chromatogram are minor impurities.
(TIF)
