# Supplementary figures and images for: Design of pH Sensitive Binding Proteins from the Hyperthermophilic Sso7d Scaffold
Source: PLoS One. 2012 Nov 7;7(11):e48928. doi: 10.1371/journal.pone.0048928 (PMC3492137; doi:10.1371/journal.pone.0048928)

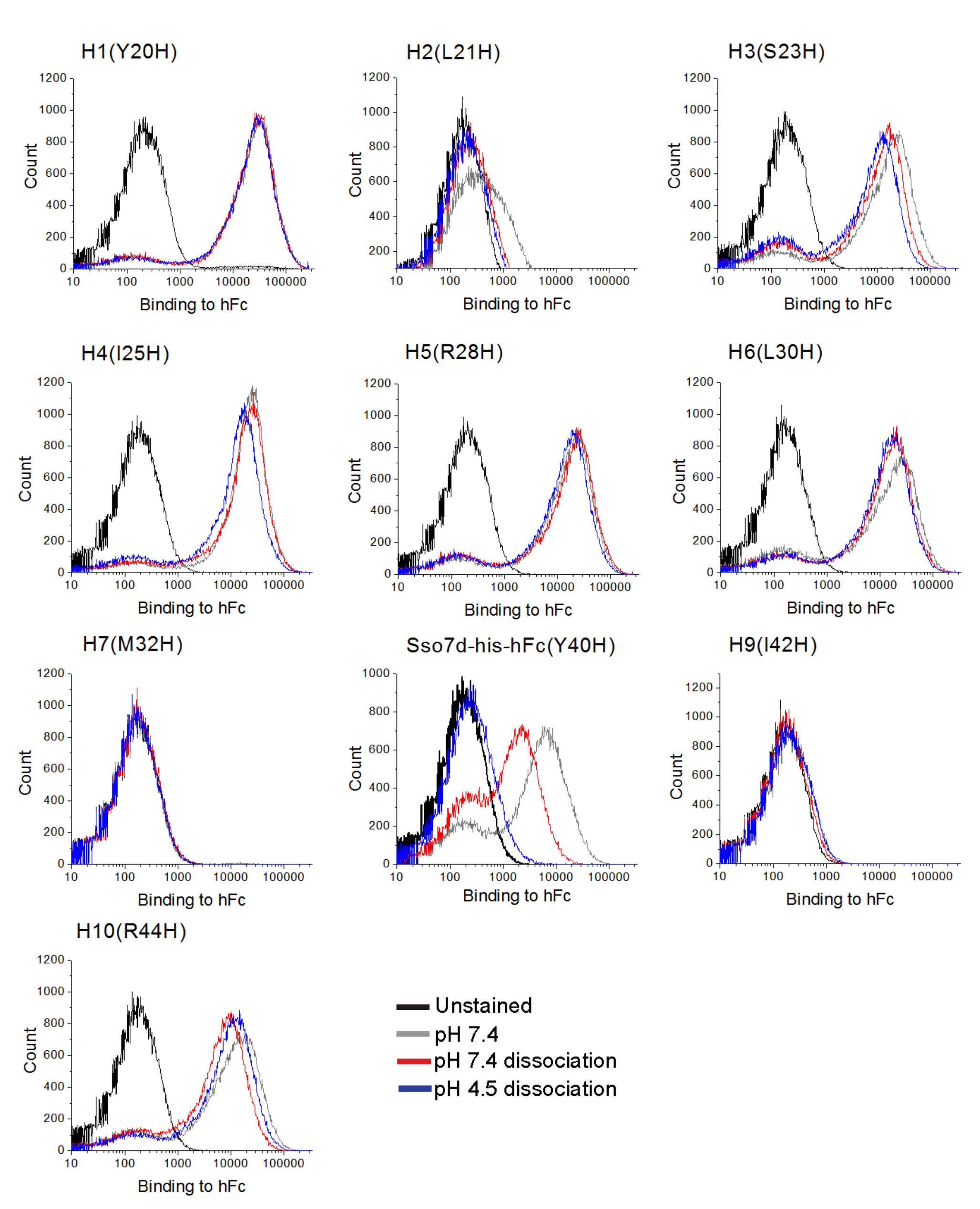

Supplement: Figure S1 — End-point assay analysis of Sso7d-hFc mutants with single histidine substitutions, to evaluate pH sensitivity. Yeast cells displaying Sso7d- hFc mutants with single histidine substitutions were incubated with 100 nM hFc-biotin and the yeast-hFc complexes were dissociated in buffers at pH 7.4 and pH 4.5. Undissociated hFc remaining on yeast surface was detected using streptavidin-phycoerythrin (strep-PE). A cell sample where no dissociation step was carried out after hFc labeling at pH 7.4, and unstained cells were used as controls. Sso7d-his-hFc was identified as a pH sensitive hFc-binder and used in further analysis. (TIF) [file pone.0048928.s001.tif]

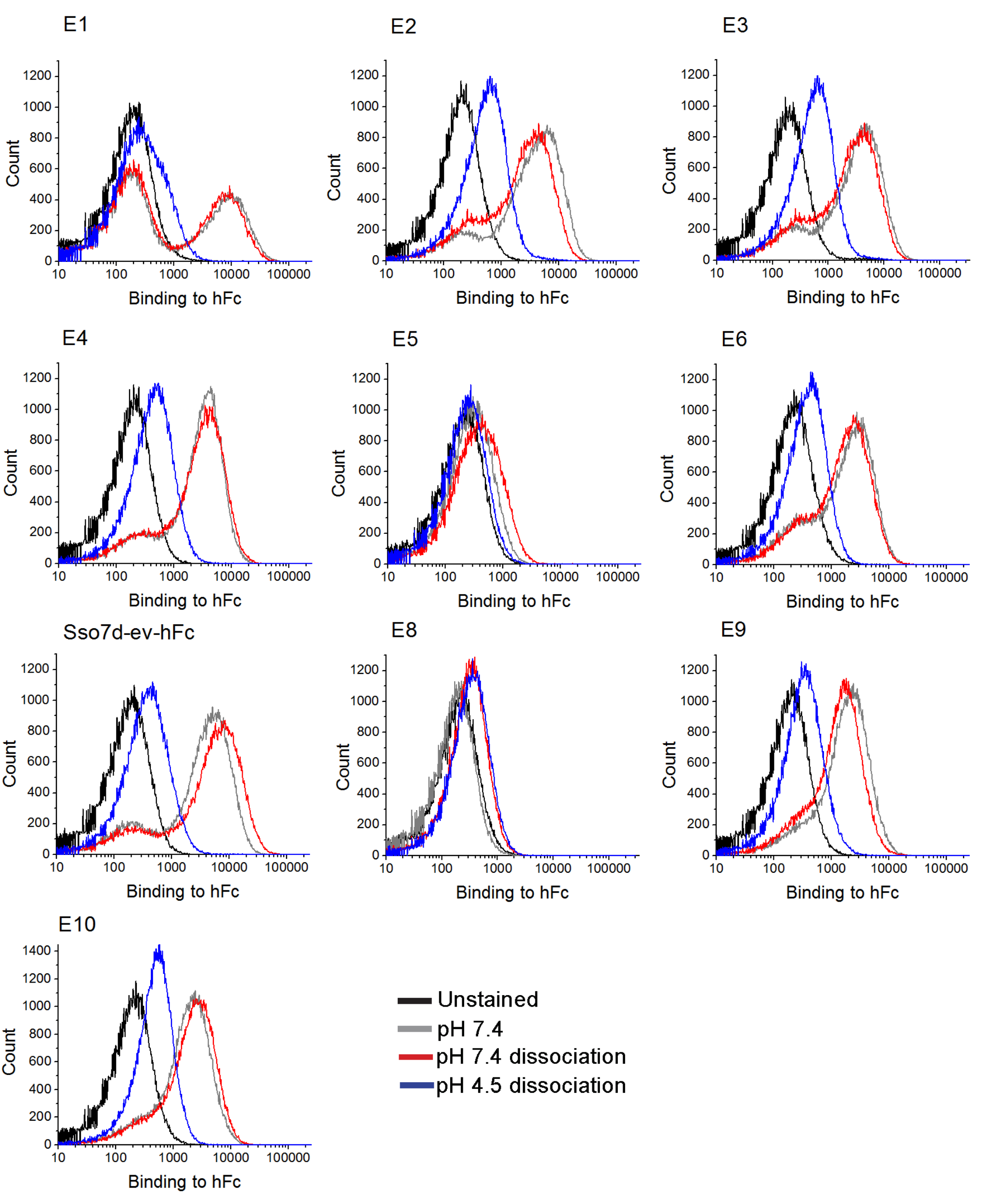

Supplement: Figure S2 — End-point assay analysis of individual clones isolated from a library generated by random mutagenesis of a pool of hFc-binders, to evaluate pH sensitivity. Yeast cells displaying pH sensitive hFc binders were incubated with 2 µM hFc-biotin and the yeast-hFc complexes were dissociated in buffers at pH 7.4 and pH 4.5. Undissociated hFc remaining on yeast surface was detected using strep-PE. A cell sample where no dissociation step was carried out after hFc labeling at pH 7.4, and unstained cells were used as controls. Sso7d-ev-hFc was identified as the best pH sensitive hFc-binder and used in further analysis. (TIF) [file pone.0048928.s002.tif]
